# Significant drop in serum C-reactive protein in patients with solid neoplasia and bacterial infection is associated with a better prognosis and identifies candidates for short-course antibiotic therapy

**DOI:** 10.1186/s12879-024-09544-1

**Published:** 2024-09-13

**Authors:** Guilherme Monteiro de Barros, Isabela Nascimento Borges, Cecilia Gómez Ravetti, Paulo Henrique Diniz, Samuel Rosa Ferreira, Lara Hemerly De Mori, Rafael Castro, Getúlio H. Okamura, Felipe Gatto, Vandack Nobre, on behalf of the “Núcleo Interdisciplinar de Investigação em Medicina Intensiva” (NIIMI), Paula Vassalo, Marcus Vinícius de Melo Andrade, Isabella Santana dos Anjos, Ronan de Sousa, Rafael Carvalho, Pablo Gustavo Oliveria, Luciana Santiago, Vitoria Rezende, Angelica Gomes, Juliana Sartorelo

**Affiliations:** 1 Graduate Program in Infectious Diseases and Tropical Medicine, School of Medicine, Universidade Federal de Minas Gerais, Av. Prof. Alfredo Balena, 190, Santa Efigênia, Belo Horizonte MG, CEP: 30130-100 Brazil; 2 Hospital das Clínicas, Universidade Federal de Minas Gerais, Av. Prof. Alfredo Balena, Santa Efigênia, Belo Horizonte MG, 190CEP: 30130-100 Brazil; 3 Department of Internal Medicine, School of Medicine, Universidade Federal de Minas Gerais, Belo Horizonte MG, Brazil

**Keywords:** Oncology, Acute bacterial infections, C-reactive protein, Antibiotic therapy

## Abstract

**Introduction:**

The greater predisposition to infections, as well as the possibility of a worse response to treatment, can lead to the excessive use of antimicrobials among cancer patients. C-reactive protein (CRP) has gained prominence as a tool for monitoring therapeutic responses and reducing the duration of antibiotic therapy; however, few studies have analyzed this protein in cancer patient populations. We hypothesize that cancer patients with a good response to antibiotic therapy show a faster decline in serum CRP levels, which would allow us to identify candidates for short-course treatments.

**Objective:**

To evaluate the behavior of serum CRP levels among adult cancer patients using antibiotic therapy, and its association with the duration of this treatment, therapeutic response, and clinical recurrence.

**Methods:**

This work consisted of a retrospective study with cancer patients admitted to a university hospital between September 2018 and December 2019. Adults (age ≥ 18 years) who underwent at least one course of antibiotic therapy were included. CRP behavior over the first 7 days of treatment was classified as: i) good response: when the CRP value on the fifth day of therapy reached 50% or less of the peak value detected in the first 48 h of treatment, and ii) poor response: Maintenance, within the same interval, of a CRP value > 50% of the peak value in the first 48 h. The duration of antibiotic therapy was categorized as up to seven full days or more. Outcomes were assessed by events that occurred during the 30 days of hospitalization or until hospital discharge. Primary outcome: Clinical recurrence of the index infection. Secondary outcomes: i) Death from any cause; ii) microbiological recurrence; iii) therapeutic response; iv) colitis associated with *Clostridioides difficile*; and v) isolation of multi-resistant bacteria, whether in clinical or surveillance samples.

**Results:**

The final analysis consisted of 212 patients, with a median age (IQ) of 59.2 (48 – 67) years old and a predominance of females (65%), who were hypertensive (35%), smokers (21%), and diabetics (17.8%). There was no difference in clinical recurrence between the two groups (8.1% vs. 12.2%; *p* = 0.364), with a lower 30-day mortality in the good CRP response group (32.2% vs. 14.5%; *p* = 0.002). Despite the tendency towards a lower occurrence of other secondary outcomes in the good response group, these differences were not statistically significant. In the poor CRP response group, outcomes like clinical recurrence, mortality, and therapeutic response were significantly worse, regardless of the duration of antibiotic treatment.

**Conclusion:**

In this study, cancer patients with a good CRP response during antibiotic therapy presented lower mortality and a higher proportion of satisfactory therapeutic responses. CRP can be a useful tool when combined with other clinical information in optimizing the duration of antimicrobial treatment in a hospitalized cancer population.

**Supplementary Information:**

The online version contains supplementary material available at 10.1186/s12879-024-09544-1.

## Introduction

The excessive use of antibiotics contributes to the emergence of multidrug-resistant bacteria, in addition to an increase in costs and the occurrence of adverse effects related to these drugs [1]. Nearly 50% of all hospitalized patients receive at least one course of antibiotic therapy during their hospital stay, with 20–30% of these prescriptions considered to be inappropriate [2]. Over the last few years, several initiatives have been launched in an attempt to promote a more rational use of these drugs, generically known by the term stewardship [3].

There is growing evidence that short courses of antibiotic therapy, equal to or shorter than seven days, are safe and may even be associated with a better prognosis, including reduced mortality and the occurrence of adverse events [4, 5]. Short-course treatments appear to be safe even in critically ill patients with more serious infectious conditions [6]. Additionally, tools that help customize antimicrobial treatment are highly desirable, as patients who have an infection at the same site may require varying durations of antibiotic therapy [7]. In this sense, the use of biomarkers, whose circulating levels reflect, with reasonable precision, the response to anti-infective treatment, associated with routine clinical and laboratory data, can help in decision-making concerning the best time to interrupt antibiotic therapy [8]. In addition to allowing more assertive decisions for each patient, this strategy has the potential to save resources, reduce selective pressure for the emergence of multi-resistant bacteria, and limit the development of adverse effects [7, 9]. Among the molecules tested for this purpose, procalcitonin and C-reactive protein (CRP) stand out, the latter being more accessible, low-cost, and with easy-to-interpret kinetics, in addition to being a marker routinely used in clinical practice [10].

Cancer patients have a greater predisposition to infections and possibly a worse response to antibiotic treatment, resulting from a multifactorial impairment of the immune response [11, 12]. Of note, most clinical trials that tested protocols based on procalcitonin or CRP to guide antibiotic therapy did not include cancer patients, especially if they were undergoing chemotherapy or were neutropenic [13–15].

Given the information reported above, this study aimed to evaluate the behavior of serum CRP levels in cancer patients using antibiotic therapy and their association with the duration of antibiotic therapy, as well as with the therapeutic response.

## Methods

### Study design and location

A retrospective study was conducted at the Hospital das Clínicas da Universidade Federal de Minas Gerais – HC-UFMG, Brazil. HC-UFMG is a teaching hospital with 465 beds and serves as a regional reference for oncology, chemotherapy, and associated complications. This study received approval from the Ethics Committee of the Universidade Federal de Minas Gerais under the number (CAAE: 39,519,720.8.0000.5149). Due to the retrospective design, the requirement for an Informed Consent Form was waived.

### Study population

Adult patients (18 years or older) diagnosed with solid cancer and who presented with a bacterial infection upon admission or during hospitalization at HC-UFMG between September 2018 and December 2019 were consecutively evaluated for potential eligibility. Inclusion criteria were: i) suspected or confirmed bacterial infection; ii) prescription of antibiotic therapy by the medical care team, maintained for at least 72 h; and iii) availability of serial CRP measurements in the first seven days of treatment. While daily doses were not mandatory, at least one dose was administered within the first 48 h of treatment, followed by two additional doses on separate days after 48 h of treatment. Patients receiving full palliative care, discharged from the hospital, or deceased within less than 48 h of antibiotic treatment, as well as high-risk febrile neutropenic patients (MASCC < 21 points), were excluded. In cases of multiple infectious episodes during the same hospitalization, only the first episode was considered for analysis purposes in this study.

The diagnosis of infection was based on the suspicion of the attending medical team during hospitalization, an inclusion indicator for the study based on clinical criteria, with or without microbiological confirmation. Cases with positive microbiological cultures from clinically relevant samples or demonstrating an infectious focus on imaging or clinical examination (e.g., skin and soft tissue infection) were considered confirmed infections. All others were considered suspected infections.

### Data collection

Clinical, imaging, and laboratory data of the included patients were obtained from electronic medical records, MV-PEP SIG SS 2013 (version 2.8.0), and the MatrixNet laboratory results system (MATRIXSAUDE, 2020). The collected data were recorded in a questionnaire designed for the present study (supplementary material), created on the Research Electronic Data Capture – Redcap platform (2004, Vanderbilt University, Nashville USA), and then exported to the SPSS statistical analysis package, version 20.0 (Armonk, NY: IBM Corp. USA).

### Study groups

Patients were categorized into two groups based on the behavior of CRP during the first seven days of antibiotic treatment, following the criteria of Borges et al., 2020: i) Good response: when the serum CRP value on the fifth day of treatment was less than or equal to 50% of the peak value detected in the first 48 h after the onset of treatment; and ii) Poor response: when the serum CRP value on the fifth day of treatment was greater than 50% of the peak value detected in the first 48 h after the onset of treatment.

Participants were then categorized into subgroups based on the duration of antibiotic therapy: i) short course of antimicrobial (≤ 7 days) and ii) long course of antimicrobial (> 7 days). Ultimately, four subgroups of patients were categorized, which were compared regarding clinical recurrence, mortality, and therapeutic response: group 1 – good biomarker response and short course of antimicrobials; group 2 – good biomarker response and long course of antimicrobials; group 3 – poor biomarker response and brief course of antimicrobials; and group 4 – poor biomarker response and long course of antimicrobials.

### Evaluated outcomes

Study outcomes were assessed at 30 days of hospitalization or at hospital discharge, whichever occurred first. The primary outcome was clinical recurrence of the infection, indicating the need to restart antibiotic therapy within 2 days. The following secondary outcomes were also assessed: i) Death from any cause; ii) Persistence or recurrence of new positive cultures for the same microorganism causing the index infection; iii) Diarrhea associated with *Clostridioides difficile*; iv) Isolation of multidrug-resistant bacteria, whether in a clinical or surveillance sample during the same hospitalization, as long as it was not the etiological agent of the index infection; and v) Length of hospital stay. Patients were also categorized as having a favorable therapeutic response when there was recovery from the infectious disease, as expected by the medical care team, or unfavorable when there was no evolution (e.g., death, unexpected clinical and/or laboratory evolution, recurrence).

### Statistical analysis

Categorical variables were expressed as percentages, while continuous variables were presented using the most appropriate measures of central tendency and dispersion for this type of distribution. Mean ± standard deviation (SD) was used for variables with a normal distribution, while medians and the interquartile range (P25%-P75%) were used for variables with a non-normal distribution. Qualitative variables were compared using the chi-squared or Fisher's exact tests, and quantitative variables were compared using the Student's t-test or Mann–Whitney U test, as indicated. A *p*-value < 0.05 was considered significant for all analyses.

## Results

**Fig. 1:**
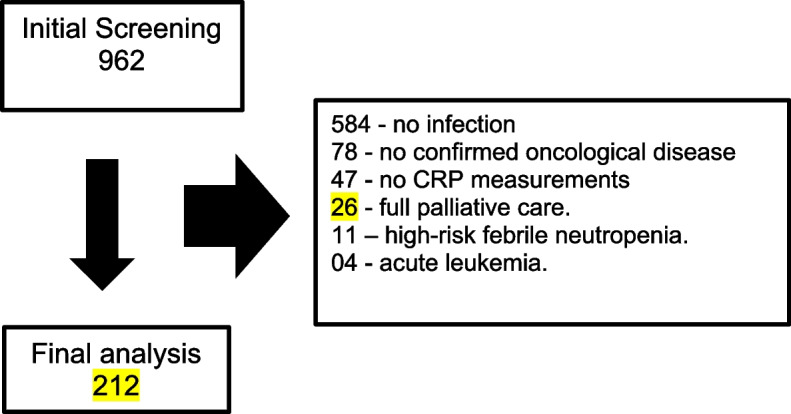
Flow chart of inclusions. CRP: C-reactive protein

**Fig. 2:**
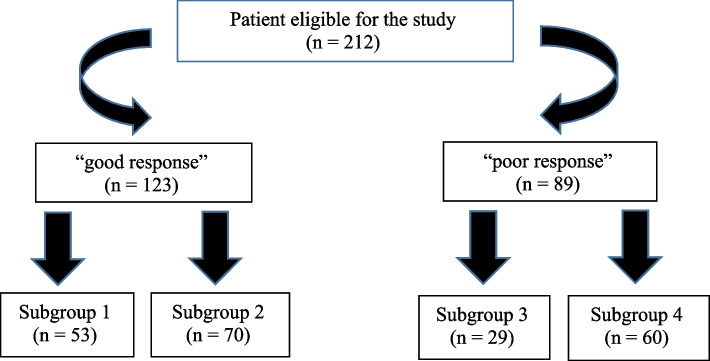
Classification of patients into groups, according to CRP response and duration of antibiotic therapy. Group 1: “good response” + use of ATB < or equal to 7 days. Group 2: “good response” + use of ATB > 7 days. Group 3: “poor response” + use of ATB < or equal to 7 days. Group 4: “poor response” + use of ATB > 7 days

During the study period, 962 patients were evaluated by the HC-UFMG clinical oncology team. Of these, 584 patients were excluded for not having a diagnosis of infection during hospitalization, 78 patients for not having a confirmed diagnosis of oncological disease, and 47 patients for a lack of CRP measurements. Four patients with hematological malignancies (acute leukemia), 11 high-risk febrile neutropenics, and 26 receiving full palliative care were also excluded. The final analysis consisted of 212 patients (Fig. 1), with groups formed according to CRP response and duration of antibiotic therapy (Fig. 2).

The main baseline characteristics of the patients included in the study are listed in Table 1, stratified by CRP response during antibiotic treatment. The most common primary sites of neoplasia were breast (22.4%), colorectal (10.3%), lung (10.3%), and non-colorectal gastrointestinal tract (9.3%). There was a predominance of patients with advanced neoplasia in stage IV (67.3%). Nearly 70% of the patients were undergoing chemotherapy at the time of admission, along with radiotherapy in 11.6% of these cases. This association was proportionally more common in the group with a poor CRP response compared to the group with a good response to this biomarker (15.5% vs. 8.9%; *p* = 0.011). A higher proportion of patients using corticosteroid therapy at an equivalent dose equal to or greater than 0.5 mg/kg/day of prednisone was observed in the group with a good CRP response (19.3% vs. 7.7%; *p* = 0.018).

**Table 1:** Main demographic and clinical characteristics of study participants, classified according to CRP response

Variables – N	Total ( *n* = 212)	Good CRP response (*n* = 123)	Poor CRP response (*n* = 89)	OR (IC95)	*P*-value
Average age	59.25 (48 – 67)	58.5 (49—66)	60 (48—67)	-	0.806
Female, in (%)	138 (64.5)	79 (63.7)	59 (65.6)	0.92 (0.53–1.6)	0.781
Hypertension, and (%)	75 (35.0)	43 (34.7)	32 (35.6)	0.96 (0.54–1.69)	0.894
Diabetes, in (%)	38 (17.8)	24 (19.4)	14 (15.6)	1.3 (0.63–2.68)	0.473
Heart failure, n (%)	8 (2.2)	6 (4.8)	2 (3.7)	2.2 (0.44–11.35)	0.319
Coronary disease, n (%)	6 (2.8)	3 (2.4)	3 (3.3)	0.71(0.14–3.64)	0.689
Chronic renal disease, n (%)	13 (6.1)	4 (3.2)	9 (10)	0.30(0.08–1.00)	0.041
Cirrhosis, in (%)	2 (0.9)	2 (1.6)	0 (0)	0.57(0.51–0.64)	0.226
COPD, and (%)	20 (9.3)	11 (8.9)	9 (10.0)	0.87 (0.34–2.21)	0.779
Smoking, and (%)	45 (21)	22 (17.7)	23 (25.6)	0.62(0.32–1.21)	0.166
Alcoholism, in (%)	21 (9.9)	9 (7.3)	12 (13.3)	0.51(0.20–1,27)	0.149
Charlson, mean (SD)	7 (6–8)	7 (6–8)	7 (6–8)	-	0.651
**Baseline neoplasm, n(%)**					
Breast	48 (22.4)	30 (24.2)	18 (20.0)	0	0
Non-colorectal GIT	21 (9.8)	13 (10.5)	8 (8.9)	0	0
Colorectal	22 (10.3)	12 (9.7)	10 (11.1)	0	0
Lung	22 (10.3)	12 (9.7)	10 (11.1)	0	0
Head and neck	16 (7.5)	9 (7.3)	7 (7.8)	0	0
Others	85 (39.7)	48 (38.7)	37 (41.1)	0	0
**Staging,** n(%)					
I	9 (4.2)	6 (4.8)	3 (3.3)	0	0
II	22 (10.3)	10 (8.1)	12 (13.3)	0	0
III	39 (18.2)	19 (15.3)	20 (22.2)	0	0
IV	144 (67.3)	89 (71.8)	55 (61.1)	0	0
**Treatment, n(%)**					
Chemotherapy (QT)	147 (68.7)	89 (71.8)	58 (64.4)	1.4(0.78–2.51)	0.254
Mono QT	61 (45.5)	35 (42.7)	26 (50.0)	0	0.407
Poly QT	73 (54.5)	47 (57.3)	26 (50.0)	0	0.407
Radiotherapy (RT)	40 (18.7)	23 (18.5)	17 (18.9)	0.97(0.48–1.96)	0.950
QT and RT	25 (11.6)	11 (8.9)	14 (15.5)	1.31(0.02–0.71)	0.011
Corticosteroid therapy	31 (14.4)	24 (19.3)	7 (7.7)	2.84(1.16–6.93)	0.018
**Purpose of oncological therapy, n(%)**					
Adjuvant	20 (9.3)	12 (9.7)	8 (8.9)	0	0
Neoadjuvant	14 (6.5)	5 (4.0)	9 (6.7)	0	0
Curative	11 (5.1)	5 (4.0)	6 (6.7)	0	0
Palliative	146 (68.2)	95 (76.6)	51 (56.7)	0	0
**Positive cultures, n(%)**	78 (36.4)	46 (37)	22 (24)	1.5(0,85 – 2,69)	0.155
Blood culture	29 (13.6)	20 (16.1)	9 (10)	1.6(0,7–3,7)	0.260
Urine culture	31 (14.5)	18 (14.5)	13 (14.4)	0	0
E. coli	28 (13.1)	16 (12.9)	12 (13.3)	0	0
K. pneumoniae	14 (6.5)	9 (7.3)	5 (5.6)	0	0
S. aureus	9 (4.2)	6 (4.8)	3 (3.3)	0	0
Community infection	166 (77.6)	99 (79.8)	67 (74.4)	1.07(0.7–1.6	0.739
Nosocomial infection	48 (22.4)	25 (20.2)	23 (25.6)	0.78(0,4–1,4	0.459
**Site of infection, n(%)**					
Pulmonary	80 (37.4)	44 (35.5)	36 (40.0)	0	0
Urinary	31 (14.5)	18 (14.5)	13 (14.4)	0	0
Skin and soft tissue	29 (13.6)	15 (12.1)	14 (15.6)	0	0
Abdominal	26 (12.1)	16 (12.9)	10 (11.1)	0	0
Not identified	23 (10.7)	15 (12.1)	8 (8.9)	0	0
**Clinical variables**					
Neutropenia (ANC < 1000), n (%)	33 (15.4)	24 (19.4)	9 (10)	1.9(0,8–4,3)	0.111
Neutrophilia, and (%)	94 (43.9)	50 (40.3)	44 (48.9)	0,82(0,5–1,3)	0.4338
Fever, no (%)	97 (45,5)	59 (47,6)	38 (42,4)	1.12(0,7–1,8)	0.632
Sepsis, n (%)	77 (36)	45 (36,3)	32 (35,6)	1.0(0,6–1,7)	0.939
Septic shock, n (%)	20 (9,3)	9 (7,2)	11 (12,2)	0.5(0,2–1,4)	0.267
**Therapeutic response, n (%)**					
Satisfactory	148 (69.2)	102 (82.3)	46 (31.1)	1.6(1,03–2,5)	0,0346
**ATB Time, median (IQR)**	-	8 (7–11)	10 (7–15)	-	0.036
**Outcomes**					
Death, n (%)	47 (22)	18 (14.5)	29 (32.2)	0.45(0,2–0,86)	0.002
Clinical Recurrence1, n (%)	21 (9.8)	10 (8.1)	11 (12.2)	0.65(0.26–1,6)	0.364
Recurrence, n (%) Microbiological2, n (%)	11 (5.1)	7 (5.6)	4 (4.4)	1.2(0,36–4,4)	0.695
Length of stay, median (IQR)	-	11.5 (8–23)	15 (8–30)	-	0.036
Colitis, n (%)	6 (2.8)	3 (2.4)	3 (3.3)	0,72(0,1–3,6)	0.689
Emergence of bacterial multidrug resistance3, n (%)	9 (4.2)	4 (3.3)	5 (5.6)	0,58(0,15–2,2)	0.416

^1^Clinical recurrence of the index infection leading to a restart of antibiotic therapy; ^2^Persistence or recurrence of positive cultures for the same microorganism causing the index infection; ^3^Isolated in a clinical or surveillance sample during the same hospitalization, as long as it was not the etiological agent of the index infection

Regarding the infectious context, there was a predominance of pulmonary focus (37.4%), followed by a urinary focus (14.5%). One third (36.3%) of the patients had microbiological confirmation of the infection. The most frequently found microorganisms were *Escherichia coli* (13.6%), *Klebsiella pneumoniae *(6.5%), and *Staphylococcus aureus* (4.2%). Criteria for sepsis were verified in 77 patients (36%) and septic shock in 20 (9.3%), with no statistical difference when comparing the groups with good and poor CRP responses. The median time of antibiotic therapy was 9 days (7–13.5), with a difference according to the behavior of the CRP, which was longer in the poor response group, 10 (7–15) vs. 8 (7–11); *p* = 0.036.

Regarding the primary outcome – clinical recurrence of infection – no difference was observed between the good and poor CRP response groups (8.1% vs. 12.2%; *p* = 0,364). Among the secondary outcomes, lower 30-day mortality was observed in the group with a good CRP response (32.2% vs. 14.5%; *p* = 0.002), as well as a higher frequency of therapeutic response considered satisfactory according to the assessment of the medical care team (82.3% vs. 31.1%; *p*  = 0,034), when compared to the poor response to this marker. Despite the tendency towards a lower occurrence of microbiological recurrence, *Clostridioides difficile* associated diarrhea, and the emergence of multidrug-resistant microorganisms in the good response group, these differences were not statistically significant.

In the analysis restricted to patients with a good CRP response (*n* = 123), when comparing patients who used up to seven days of antibiotic therapy with those who received longer courses of treatment, a higher proportion of clinical recurrence (9.4% vs. 7.1%, *p* = 0.134), a lower proportion of deaths (3.2% vs. 19.5%, *p* < 0.001), a higher proportion of favorable therapeutic response (3.2% vs. 26.4%; *p* < 0.001), and a lower proportion of microbiological recurrence (0 vs. 10%, *p* < 0.134) were observed in the group that received a short course of antibiotic therapy. In this subgroup, findings related to clinical recurrence (9.4% vs. 7.1%, *p* < 0.134) and microbiological recurrence (0 vs. 10%, *p* < 0.134) showed no significant differences.

Among patients with a poor CRP response (*n* = 89), when comparing those who used up to seven days of antibiotic therapy with those who received longer courses of treatment, there were: a lower number of deaths (27.6% vs. 35%), and a higher proportion of therapeutic response considered favorable (62.1% vs. 45%), and similar number of clinical recurrence events (13.8% vs. 11.7%), and microbiological recurrence (0% vs. 6.7%;) (Table 2).

**Table 2:** Primary and secondary outcomes between subgroups

Outcomes n (%)	Subgroup 1 Good CRP response and short ATB course	Subgroup 2 Good PCR response and long course of ATB	Subgroup 3 Poor PCR response and short course of ATB	Subgroup 4 Poor PCR response and long course of ATB	*P*-value
**Total**	53	70	29	60	
Mortality	2 (3.8)	16 (22.9)	8 (27.6)	21 (35)	< 0.001*
Favorable therapeutic response	51 (96.2)	50 (71.4.)	18 (62.1)	27 (45)	< 0.001*
Clinical recurrence	5 (9.4)	5 (7.1)	4 (13.8)	7 (11.7)	0.134
Microbiological recurrence	0(0)	7 (10)	0	4 (6.7)	0.813

^*^ Indicate the *p*-value between subgroups 1, 3, and 4

**Fig. 3:**
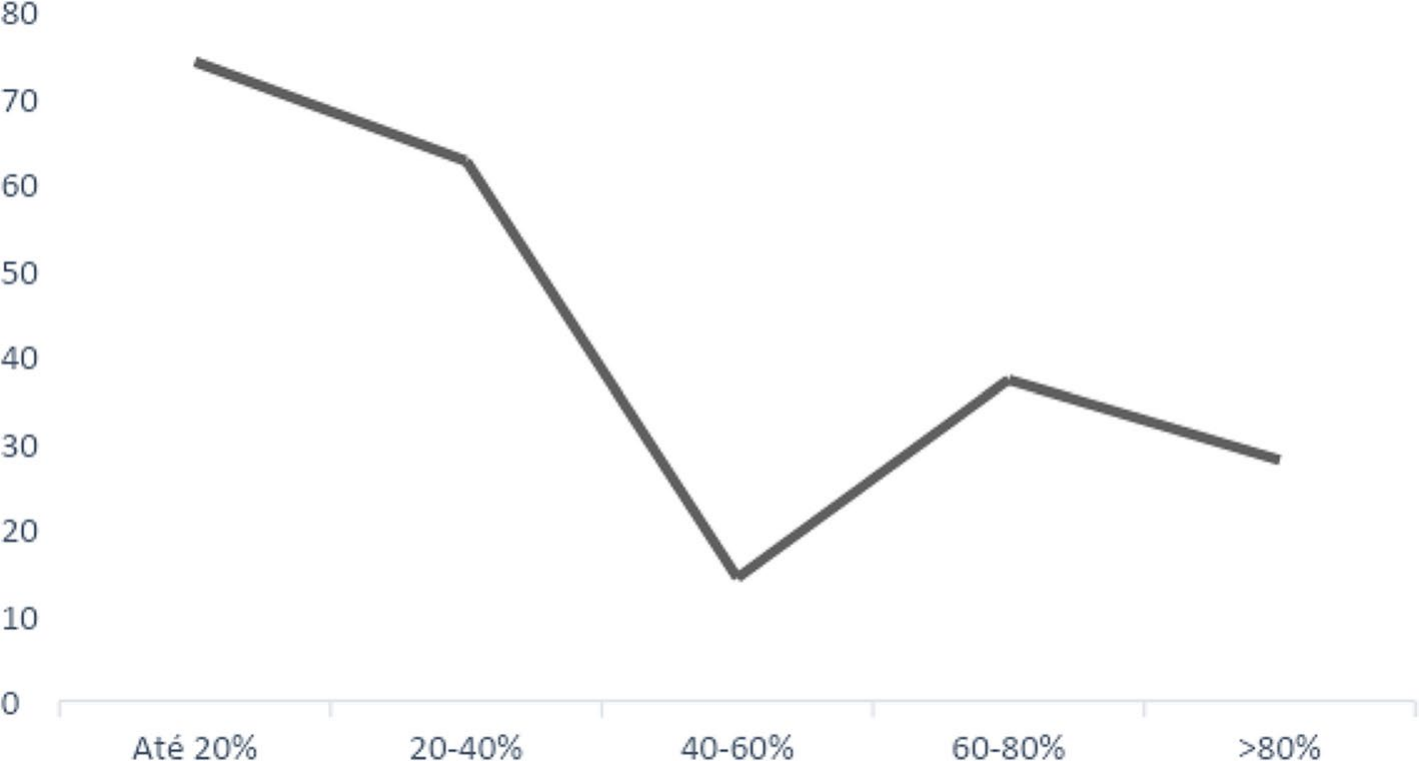
Hospital mortality (%) according to the strata of CRP decline

When hospital mortality was studied, stratified into groups according to the percentage of decrease in CRP on the 5th day of antibiotic therapy (decrease of up to 20%; 20 to 40%; 40 to 60%; 60 to 80%; and above 80% in serum levels), there was a clear trend indicating that the greater the decline in CRP, the higher the probability of a decrease in mortality (Fig. 3).

## Discussion

The present study revealed that in patients with oncological diseases hospitalized with acute bacterial infections, the behavior of circulating CRP levels could be useful in identifying those with better clinical outcomes, regardless of the duration of antibiotic treatment. Lower hospital mortality was observed in those with a good CRP response to antibiotic therapy. Interestingly, mortality proved to be higher among those who received longer courses of treatment (i.e., more than 7 days of antibiotic therapy). These findings are similar to those observed in non-oncological populations, including critically ill patients [16, 17]. In the multicenter randomized clinical trial conducted by Von Dach and collaborators, the researchers aimed to determine whether a shorter, fixed duration of antibiotic treatment for Gram-negative bacteremia could be as effective as the traditional 14-day course. The study found that the fixed, shorter duration of antibiotic treatment was not inferior [18].

In a prospective cohort of critically ill patients with active neoplasia hospitalized for pneumonia, Rabello and colleagues observed that among surviving patients, there was a higher frequency of a rapid response to CRP (decrease > 60% on the fourth day of therapy) when compared to non-survivors [19]. El Haddad and collaborators, in a post-hoc analysis of a prospective cohort using PCT as a biomarker, found no significant differences in mortality and infectious recurrence in patients with good biomarker response criteria regardless of the duration of antimicrobial use (less than or equal to 7 days vs. greater than 7 days). These findings are similar to ours, although based on a different biomarker [20]. The favorable data regarding CRP shed light on the possibility of using a more accessible and low-cost biomarker. Oliveira and collaborators carried out a randomized clinical trial to compare the effectiveness of two strategies of antibiotic therapy based on biomarkers kinetics in septic patients: one based on serum PCT levels and the other based on serum CRP levels. These authors found no superiority of the PCT-based protocol over the CRP-based protocol in the management of septic patients [21].

In our study, among patients with a good CRP response during antibiotic treatment, those who used longer courses of treatment had higher rates of mortality compared to those who received up to seven days of treatment. The use of long-course antimicrobial therapies may be associated with adverse outcomes without the benefit of lower mortality [22]. Kenjikubo and collaborators conducted a systematic review and meta-analysis to investigate the optimal duration of antibiotic therapy and its impact on clinical outcomes in patients with bloodstream infections. Their findings revealed that shorter courses of antibiotic therapy (ranging from 7 to 10 days) were generally not inferior to longer courses. Additionally, shorter courses can be associated with a potential reduction in antibiotic-related adverse events and a lower risk of developing antibiotic resistance [23].

It is worth noting that in patients with a poor CRP response, there was a tendency toward worse outcomes, including clinical recurrence, mortality, and therapeutic response, regardless of the duration of antibiotic treatment. In other words, prolonging therapy with these drugs does not result in benefits in this population. The persistence of high serum CRP levels can be explained by factors other than the persistence of active infection and was associated with unfavorable clinical evolution in other studies [19]. Inflammation plays a significant role in cancer progression and response to treatment. Biomarkers of systemic inflammation, such as CRP, can be associated with oncologic prognosis and treatment outcomes [24]. Among oncologic patients, it is important to look for other mechanisms that explain the persistence of high levels of the marker, through careful reassessment of the clinical condition in question.

This study contains some limitations that must be observed. First, this is a retrospective study, which increases the possibility of error when obtaining data. However, the findings are in line with the results of randomized clinical trials on the topic [13, 18], which suggests a good quality of the information obtained and attests to the validity of our findings. Second, the sample size studied was relatively small, and a convenience sample, which limits the power of our statistical inferences and conclusions. Even so, the consistency of the findings allows us to consider the study sufficient to support the proposition of additional studies, such as a clinical trial to test an antibiotic therapy strategy based on the behavior of CRP in cancer patients hospitalized with acute bacterial infection. Third, the definition of infection and therapeutic response were obtained based on electronic record evaluation and are subject to subjective impressions and/or recording failures. To limit the negative influence of these aspects, we sought to standardize the definitions of all variables before data collection, especially the outcomes.

## Conclusion

Serial CRP evaluation among cancer patients hospitalized with acute bacterial infection can help pinpoint individuals with a good therapeutic response and potentially identify candidates for shorter, more individualized antibiotic therapy. In patients with persistently elevated serum CRP levels, prolonged antibiotic therapy appears to offer no benefit in most cases. Larger studies with a prospective design are warranted to further clarify the role of biomarkers in oncology patient populations.

## Supplementary Information

Supplementary Material 1.

## Data Availability

The datasets used and/or analysed during the current study are available from the corresponding author on reasonable request.

## Abbreviations

CRP: C-reactive protein
ECDC/EMEA: European Centre for Disease Prevention and Control/European Medicines Agency
HC-UFMG: Hospital das Clínicas da Universidade Federal de Minas Gerais
IQ: Interquartile
MASCC: Multinational Association for Supportive Care in Cancer
PCT: Procalcitonin
SD: Standard Deviation
SPSS: Statistical Package for the Social Sciences
WHO: World Health Organization
