# Supplementary material for: Significant drop in serum C-reactive protein in patients with solid neoplasia and bacterial infection is associated with a better prognosis and identifies candidates for short-course antibiotic therapy
Source: BMC Infect Dis. 2024 Sep 13;24:974. doi: 10.1186/s12879-024-09544-1 (PMC11401243; doi:10.1186/s12879-024-09544-1)
Supplement: Supplementary file 1 — Supplementary Material 1. [file 12879_2024_9544_MOESM1_ESM.docx]

Questionnaire designed for “Significant drop in serum C-reactive protein in patients with solid neoplasia and bacterial infection is associated with a better prognosis and identifies candidates for short-course antibiotic therapy”

Identification

1. Study ID
2. Record
3. Date of enrollment in the study
4. Name
5. Date of birth
6. Age (years)
7. Sex
8. Charlson Comorbidity Index

Comorbidities (No/Yes)

1. Hypertension?
2. Diabetes?
3. Heart failure with reduced EF? (LVEF No ≤40%)?
4. Coronary artery disease?
5. Chronic kidney disease?
6. Chronic liver disease?
7. Chronic obstructive pulmonary disease?
8. Smoking?
9. Alcoholism?

Hospitalization Data

1. Date of admission
2. Date of start of antibiotic therapy
3. Admission to ICU? (No /Yes)
4. ICU stay duration

Epidemiology of Neoplasia

1. Year of diagnosis
2. Location: (Breast; Ovary; Prostate; Lung; Urothelial and Kidneys; Pancreas; Skin; Hidden; Soft Tissues; Esophagus; Uterus; Germ Cells; Colorectal; Head and Neck; Non-colorectal Gastrointestinal; Hematopoietic Tissue; Central Nervous System.
3. Histological Type: (Adenocarcinoma; Other; Carcinomas; Sarcoma; Histiocytoma; Carcinosarcoma; Squamous Cell Carcinoma, Invasive Ductal Carcinoma, Lobular Carcinoma; Poorly Differentiated Carcinoma; Non-small Cell Lung Carcinoma, Soft Tissue Sarcoma, Melanoma).
4. Staging: ( I "Local"; II "Locally Advanced"; III "Lymph Node Invasion"; IV "Metastatic")
5. Disease Activity: (Remission (> 3 months without CT and without evidence of measurable disease). Active (in CT or less than 3 months of CT). Recurrence (return of the disease at least after 3 months without treatment and without evidence of disease). Progression (objective increase in volume and/or disease sites).
6. Purpose of treatment: ( Adjuvant: treatment after complete resection of the lesion, in the absence of measurable residual disease, usually lasting 3-6 months); Neoadjuvant (prior): before surgery, with proposed downstaging of the disease; Curative (germ cell tumors): for example, testicular, germ cell ovarian-, and high-grade lymphomas); Palliative: (disease without possibility of cure usually when there is metastasis or recurrence of the disease).

Specific Cancer Treatment

1. Chemotherapy? No Yes
2. Regimen Type: ( Monotherapy; Combination Therapy)
3. Drugs Used: Platinum (Cisplatin, Carboplatin...); Anthracyclines (Doxorubicin, Epirubicin...); Taxanes (Docetaxel, Paclitaxel...); Alkylating Agents (Ifosfamide, Cyclophosphamide); (FOLFOX, FLOX, bFOL...); Oxaliplatin + Fluorouracil + leucovorin. (FOLFIRI, IFL...) Irinotecan + Fluorouracil +leucovorin. FOLFIRINOX – Oxaliplatin + Irinotecan; Fluorouracil + leucovorin or mFOLFIRINOX; Others; Not found
4. Radiation Therapy? No/Yes
5. Time since last RT session: (Less than or equal to 8 weeks (RT effect)); (More than 8 weeks.)
6. Concomitant with CT? No/ Yes
7. Corticosteroid Therapy? No Yes ( Equivalent dose of prednisone. < 0.5mg/kg/day; > 0.5 < 1mg/kg/day or > 1.1mg/kg/day.
8. Biological Immunotherapy? No Yes

Characteristics of the Infectious Context

1. Criteria: Clinical; Clinical + Microbiological Confirmation
2. Community-Acquired Infection or Healthcare-Associated Infectious
3. Site of infection: Abdominal; Bloodstream; Device; Endocarditis; Soft Tissue; Pulmonary; Central Nervous System; Urinary; Unidentified; Surgical Wound; Biliary; Tumor (Tumor Necrosis)
4. Positive Cultures?: No/Yes
5. Sample: Blood Culture; Urinary Culture; Tracheal Aspiration; Tissue Culture; Other.
6. Isolated Microorganism: Escherichia coli; Proteus mirabilis; Polymicrobial; Enterococcus faecalis; Corynebacterium sp; Klebsiella pneumoniae; Staphylococcus aureus; Streptococcus pyogenes; Streptococcus pneumoniae; Pseudomonas aeruginosa; Enterobacter sp; Citrobacter freundii; Acinetobacter baumannii; Other
7. Resistance Profile: MDR: "Antimicrobial resistance shown by a species of microorganism to at least one antimicrobial drug in three or more antimicrobial categories"; Not Applicable; Sensitive; ESBL; MRSA; Multidrug-resistant (MDR) VRE.
8. Neutropenia? No/ Yes:
9. Neutropenia Range (< 100); (100 – 500) (501 – 1000)
10. Colony-Stimulating Factor Use? No/ Yes
11. Fever? No/Yes
12. Neutrophilia? No (Values above LSN) Yes
13. Sepsis? (Sepsis 3) No Yes
14. Septic Shock? (Sepsis 3) No Yes
15. SOFA in “First day of antibiotic”
16. Antibiotic Use? No Yes (Piperacillin + tazobactam; Meropenem; Clavulin; Azithromycin; Clarithromycin; Cefepime; Metronidazole; Ampicillin + Sulbactam; Oxacillin; Ampicillin; Vancomycin; Ceftriaxone; Ciprofloxacin; Tigecycline; Polymyxins; Cephalexin; Gentamicin; Teicoplanin; Amikacin; Trimethoprim/Sulfamethoxazole; Ceftazidime;
17. Antibiotic Therapy Duration (days)
18. Was the Clinical Outcome of the infection management judged as satisfactory by the assisting team? No/ Yes /Partially

C Reactive Protein

(PCT on admission) PCT D1

(PCT day 1) PCT D2

(PCT day 2) PCT D3

(PCT day 3) PCT D4

(PCT day 4) PCT D5

(PCT day 5) PCT D6

(PCT day 6) PCT D7

(PCT day 7)

Primary Outcomes:

1. Clinical recurrence of the index infection? Yes/No (As defined by the assisting team, leading to restart of antibiotic therapy.)

Secondary Outcomes:

1. Therapeutic failure over 30 days of hospitalization.
2. Death? Yes No
3. Persistence or new positive cultures? Yes No (Identified the same causative microorganism of the index infection.)
4. Clostridium difficile-associated Colitis? Yes/ No
5. Emergence of multidrug-resistant microorganism? Yes/ No (Isolated in clinical or surveillance samples during the same hospitalization, provided it was not the etiological agent of the index infection.)
6. Length of Hospital Stay (In days.)
